# CT‐based Morphometric Analysis of Approach of Percutaneous Transforaminal Endoscopic Lumbar Interbody Fusion

**DOI:** 10.1111/os.12434

**Published:** 2019-03-21

**Authors:** Kai‐hui Zhang, Wei‐hao Zhang, Bao‐shan Xu, Xiao‐man Dong, Lin Guo, Li‐long Du, Hai‐wei Xu

**Affiliations:** ^1^ Graduate School of Tianjin Medical University Tianjin China; ^2^ Department of Minimally Invasive Spine Surgery Tianjin Hospital Tianjin China; ^3^ Department of Radiology Tianjin Hospital Tianjin China

**Keywords:** Computed tomography, Endoscopy, Interbody fusion cage, Nerve root, Spinal fusion

## Abstract

**Objectives:**

A radiographic study was designed to measure the relationship of the exiting nerve root and its surroundings to the corresponding intervertebral disc for percutaneous transforaminal endoscopic lumbar interbody fusion to better understand the regional anatomy and to improve clinical applications.

**Methods:**

A retrospective study from January 2017 to October 2017 was conducted at Tianjin Hospital. CT images were obtained from patients presenting low back pain (110 patients), and analysis was performed bilaterally from L_2‐3_ to L_5_S_1_. In the rotating coronal plane we analyzed: the nerve root–dural sac distance at the superior and inferior margins of the disc (Js, Ji); the nerve root–pedicle distance at the medial, middle, and lateral borders of the pedicle (Pa, Pb, Pc); the pedicle width (W); and the safe working zone, defined as a trapezoid bounded by the inferior pedicle and the exiting nerve root (S). In the transverse plane, the nerve root‐articular process and the shortest distance for the nerve root‐articular process joint surface were analyzed at the superior and inferior margins of the disc (Gs, Gi), respectively. The groups were analyzed using ANOVA, and paired *t*‐tests were used to compare the left and right sides.

**Results:**

From L_2‐3_ to L_5_S_1_, the distance of the nerve root to the dural sac was larger at the inferior margin of the disc. From L_2‐3_ to L_5_S_1_, each segment of the vertebral nerve root‐pedicle distance gradually decreased from medial to lateral. From L_2‐3_ to L_5_S_1_, the distance from the exiting nerve root to the middle and lateral margins of the pedicle gradually decreased, with L_5_S_1_ being the minimum. Some significant differences were observed between the left and right sides for L_4‐5_ and L_5_S_1_. The pedicle width of the vertebral body and the mean area for the safe working zone gradually increased from L_2‐3_ to L_5_S_1_. In the axial plane, the shortest distance between the nerve root and articular process joint surface at the inferior margin of the disc was greater than the distance for the nerve root to the articular process at the superior margin of the disc from L_2‐3_ to L_5_S_1_. There were no significant differences between the two sides.

**Conclusions:**

It is more difficult to implant a cage with a width of 10 mm above the L_3‐4_ level. By removing part of the superior articular process, the safe working area can be expanded, and damage to the nerve or other structures can be avoided when implanting a cage.

## Introduction

Lumbar degenerative diseases are common clinical diseases. Spinal fusion can increase the stability of the spine, thereby alleviating pain, restoring function, and improving the quality of life of patients^1^. In 1911, Albee^2^ was the first to use tibia bones to increase the stability of the spine in animal experiments; then, Hibbs^3^ reported a spinal fusion technique for interbody fusion. However, based on the biomechanical load distribution characteristics of the lumbar vertebrae, the primary mechanical load is distributed throughout the vertebral body of the lumbar vertebrae. Due to the distribution of biomechanical load throughout the lumbar vertebrae, fusion of the lumbar vertebrae is a better method for improving the stability of the spine^4^.

Until now, approaches for lumbar interbody fusion have primarily included anterior lumbar interbody fusion (ALIF), posterior lumbar interbody fusion (PLIF), transforaminal lumbar interbody fusion (TLIF), extreme lateral interbody fusion (XLIF), minimally invasive transforaminal lumbar interbody fusion (MIS‐TLIF), oblique lumbar interbody fusion (OLIF), axial lumbar intervertebral fusion (AxiaLIF) and percutaneous endoscopic lumbar interbody fusion. In 1948, Lane and Moore^5^ first reported the use of ALIF for the treatment of degenerative lumbar disease, which is conducive to improving the stability of the middle spinal column. PLIF was proposed by Croward^6^ in 1940 and is currently widely used for lumbar interbody fusion. The advantage of PLIF lies in the direct visualization of the relevant anatomy and the ability to access most areas of the intervertebral disc, but this approach can result in pain, a longer recovery time, and other morbidities due to the use of open surgery. TLIF is a new method for reaching the intervertebral disc through a posterolateral approach of the intervertebral foramen and was reported in 1982 by Harms and Rolinger^7^; this method can reduce the traction of the dural sac and nerve roots compared to PLIF. MIS‐TLIF was proposed by Foley *et al*.^8^ to reduce tissue damage while also achieving adequate decompression and favorable fusion during the approach; the incision and muscle injury are significantly reduced compared to those of conventional open surgery. However, MIS‐TLIF still results in considerable bleeding and more serious complications. XLIF or lateral lumbar interbody fusion (LLIF) was first described by Ozgur *et al*.^9^ in 2006; the approach is performed from the retroperitoneal space to the intervertebral space. With this method, the operative time, hospital stay, and recovery time are significantly better than those for open surgery. However, it is easy to damage the lumbosacral plexus and it is difficult to treat the L_5_S_1_ segment, as well as the L_2‐3_ segment and above, because these regions are blocked by the ribs. OLIF, as proposed by Silvestre^10^ in 2012, is an anterior lateral retroperitoneal approach that passes through the natural passage between the psoas muscles and vessels in front of the vertebral body to avoid damaging the psoas muscle and achieve better clinical efficacy. AxiaLIF was first reported by Craig^11^. This approach is better for the anterior, posterior, and lateral structures of the spine. However, these traditional open lumbar interbody fusions have limitations. These methods can cause more damage to the small joints, paravertebral muscles, and soft tissues, which may lead to problems such as weakening of the back muscles and chronic lower back pain. In recent years, with the development of medical devices and progress in surgical techniques, endoscopic methods for the spine have made revolutionary progress^12^, with the characteristics of minimal surgical trauma and precise surgical results.

Percutaneous endoscopic lumbar interbody fusion is based on the minimally invasive percutaneous transforaminal (PTED) technique, which has recently been widely used in the treatment of lumbar disc herniation. The minimally invasive fusion procedure is performed through an endoscopic transforaminal approach to the target intervertebral disc. The resulting smaller incision, minor trauma, and faster recovery have attracted the attention of spine surgeons^13^. The surgical procedure includes a percutaneous puncture to locate the target segment, step‐by‐step expansion, placement of the operation channel for decompression, endplate preparation, implantation of the interbody cage, and fixation of a percutaneous pedicle screw^14^. This surgical approach reduces the damage to the posterior spinal canal, making it less traumatic than traditional open surgery, with less bleeding, faster postoperative recovery, and a shorter hospital stay.

However, the procedure requires the surgeon to have experience in endoscopic surgery. The operating area is the Kambin triangle^15^, which is bounded anteriorly by the exiting nerve root, inferiorly by the endplate of the lower vertebrae, posteriorly by the superior articular process, and medially by the traversing nerve root. Operating in this area can minimize damage to the exiting nerve root. However, this area may have intervertebral foramen stenosis, a high iliac crest, obstructions of the superior articular process, and other related factors, resulting in a small space for the endoscopic operation. In these cases, improper operation or a larger cage can easily damage the exiting nerve root and influence the final recovery of the patients.

Therefore, this article provides a CT‐based morphometric analysis of the approach of full endoscopic lumbar interbody fusion. The aim of the present study was to provide surgeons with: (i) a better understanding of the anatomical structure around the nerve roots in the intervertebral foramen; and (ii) a reference for the size of the interbody fusion cage in this procedure.

## Materials and Methods

This study was a retrospective study and was approved by the Tianjin Hospital ethics committee. Patients were recruited who had reported low back pain from January to October 2017 in Tianjin Hospital. The inclusion criteria are as follows: (i) patients over the age of 20 with low back pain; (ii) patients received CT scan; and (iii) patients with no previous spine surgery, gross deformity, lumbosacral transitional vertebrae, scoliosis, bone hyperplasia, intervertebral disc herniation, or intervertebral space collapse. The exclusion criteria are as follows: spinal inflammation, tuberculosis, tumor, trauma, and bone disease. CT images (GE Discovery CT750 HD Scanner, USA) were acquired in the standard supine position (120 kV, 100 mA, 1.0‐s duration, 20‐cm field of view, 512 × 512 matrix). The scan was acquired in the spiral scan mode, with the scan baseline parallel to the vertebral body. The scanning range is from L_1_ to S_3_, and the scanning layer thickness is 3 mm, with a spacing of 3 mm, tube current of 220 mA, and tube voltage of 120 kV. Image postprocessing and related measurements were performed using a professional image workstation (AW46, GE, USA).

### *Morphological Parameters*

For the CT images of the patients, we selected the L_2‐3_ to L_5_S_1_ levels and analyzed the relevant values on both sides. The data were measured by two senior doctors at the Department of Radiology of Tianjin Hospital on an AW46 image workstation, and the average value was taken.

### *Rotating Coronal Plane*

**Figure 1:**
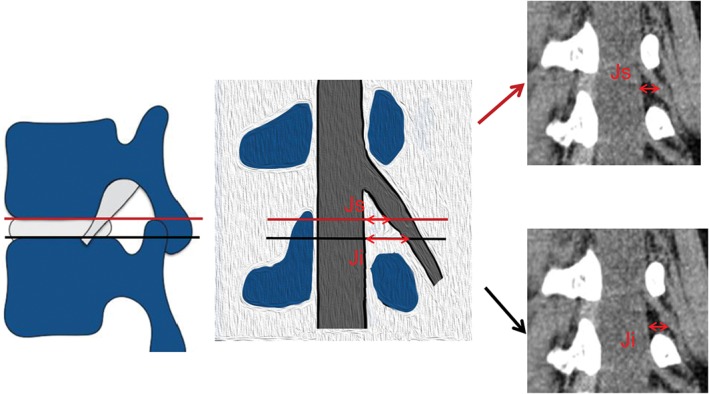
Example of the parameters Js and Ji on the rotating coronal plane of the superior and inferior margins of the target intervertebral disc. (Js, the distance from the exiting nerve root to the dural sac on the superior margin of the intervertebral disc; Ji, the distance from the exiting nerve root to the dural sac on the inferior margin of the intervertebral disc.)

**Figure 2:**
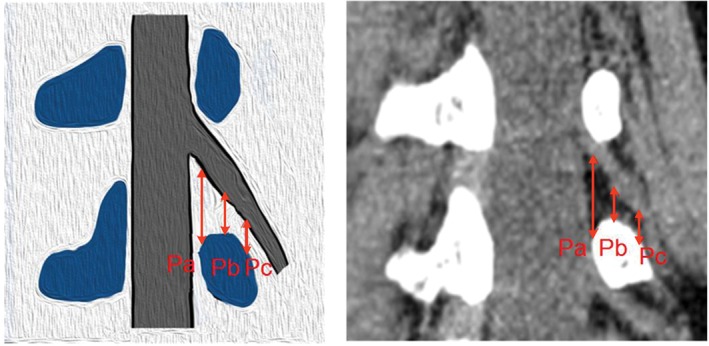
Example of the parameters Pa, Pb, and Pc on the rotating coronal plane. (Pa, the distance from the exiting nerve root to the medial border of the pedicle; Pb, the distance from the exiting nerve root to the middle border of the pedicle; Pc, the distance from the exiting nerve root to the lateral border of the pedicle.)

**Figure 3:**
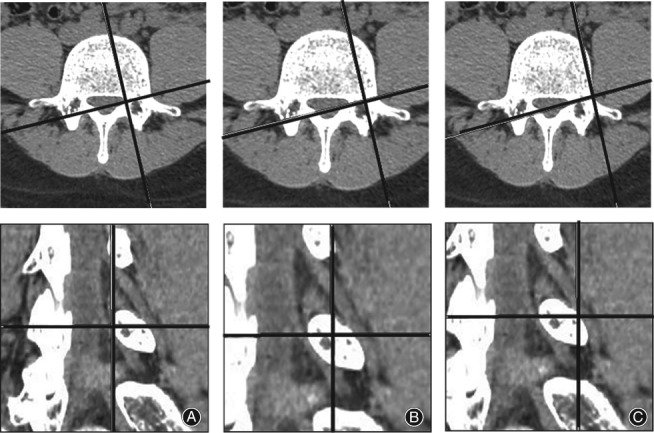
Examples of the medial edge of the pedicle (A), the middle of the pedicle (B), and the lateral edge of the pedicle (C), as located on axial and coronal CT images.

**Figure 4:**
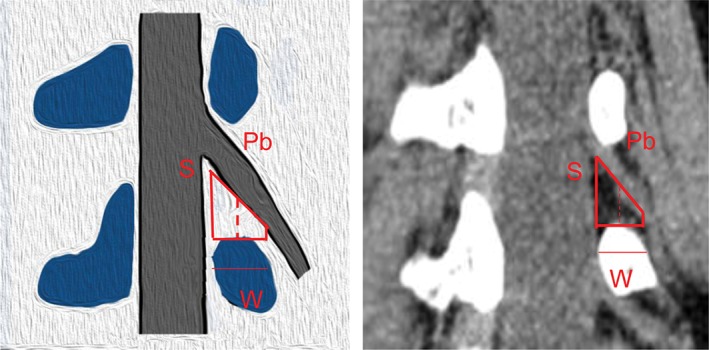
Example of the parameter S on the rotating coronal plane. (W, the pedicle width; S, safe working area, which defined as the trapezoid bounded by the inferior pedicle and the exiting nerve root. S = Pb*W.)

We selected a rotating coronal plane (the vertical plane of the long axis of the pedicle through which the exiting nerve root is visible) using the image workstation. We obtained the following measurements. First, locating the superior and inferior margins of the intervertebral disc in coronal and sagittal images, the distance between the exiting nerve root and the dural sac was measured on the superior margins of the intervertebral disc on the rotating coronal plane (Js), as was the distance from the exiting nerve root to the dural sac on the inferior margin of the intervertebral disc (Ji), as shown in Fig. 1. Second, in the axial image, the medial, middle, and lateral points of the pedicle were located for the rotating coronal plane, as shown in Figs 2 and 3. The vertical distance (Pa) from the exiting nerve root to the medial border of the pedicle was measured, as was the vertical distance (Pb) from the exiting nerve root to the middle border of the pedicle and the vertical distance (Pc) from the exiting nerve root to the lateral border of the pedicle. (iii) In the rotating coronal plane, we measured the pedicle width (W) and safe working area (S), defined as the trapezoid bounded by the inferior pedicle and the exiting nerve root (S = Pb*W), as shown in Fig. 4.

### *Axis Plane*

**Figure 5:**
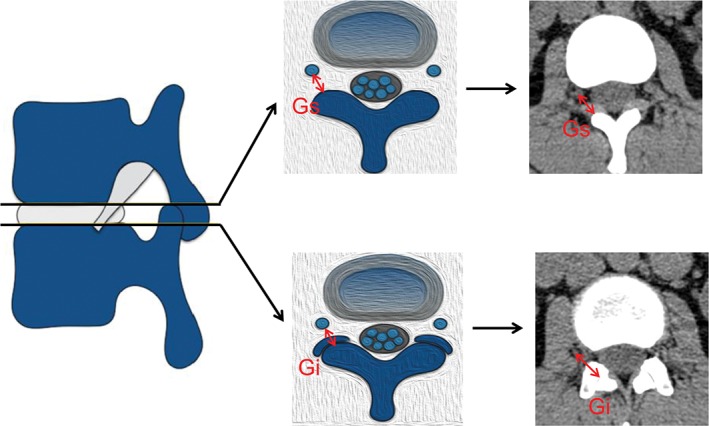
Example of the parameters Gs and Gi on the axis plane. (Gs, the shortest distance from the exiting nerve root to the articular process on the superior margin of the disc; Gi, the shortest distance from the exiting nerve root to the facet joint surface at the inferior margin of the disc.)

The axis image of the intervertebral disc was selected for measurement analysis (Fig. 5). Measurements were acquired for: (i) the shortest distance (Gs) from the exiting nerve root to the articular process on the superior margin of the disc; and (ii) the shortest distance from the exiting nerve root to the facet joint surface on the inferior margin of the disc (Gi).

### *Statistical Methods*

The data in this study were collected by two senior doctors in the Department of Radiology at Tianjin Hospital. The reliability of the data measured by the two doctors was determined using the intraclass correlation coefficient (ICC). An ICC <0.4 indicates poor data consistency, while an ICC >0.75 indicates better data consistency. All data were analyzed using the SPSS 20.0 statistical software (IBM, USA). The mean values and standard deviations were calculated, and one‐way ANOVA was used to determine whether differences were statistically significant. The paired *t*‐test was used to compare the left and right sides, where *P* < 0.05 was considered statistically significant.

## Result

Following the inclusion and exclusion criteria, we recruited 110 patients (20–79 years old, mean age 45.6 years; 49 males, 61 females) with CT images reporting low back pain from January to October 2017. The average values for the two planes are summarized in Tables 1, 2, 3. The ICC values for the parameters in both planes ranged from 0.796 to 0.916, indicating a good intragroup consistency for the measured data.

**Table 1:** Measurement on the rotating coronal plane (1) (mm, $\bar{x}$ *± s*)

Level/Side	Js	Ji	Pa	Pb	Pc
L_2‐3_					
Two‐sided	6.71 ± 2.10	11.89 ± 2.55	18.62 ± 2.50	9.05 ± 1.82	7.15 ± 2.27
Left	6.78 ± 2.09	11.97 ± 2.45	18.80 ± 2.39	9.13 ± 1.72	7.33 ± 2.17
Right	6.65 ± 2.12	11.82 ± 2.66	18.45 ± 2.61	8.97 ± 1.93	6.96 ± 2.36
L_3‐4_					
Two‐sided	7.10 ± 2.24	12.17 ± 2.62	19.56 ± 2.75	7.92 ± 1.93*	4.69 ± 2.39*
Left	7.08 ± 2.02	12.27 ± 2.59	19.80 ± 2.88	8.03 ± 2.01	4.71 ± 2.29
Right	7.12 ± 2.44	12.08 ± 2.66	19.32 ± 2.61	7.80 ± 1.86	4.66 ± 2.49
L_4‐5_					
Two‐sided	10.27 ± 2.82†	17.97 ± 3.50†	19.63 ± 2.40	7.81 ± 2.14†	5.02 ± 2.31†
Left	10.41 ± 2.83	17.72 ± 3.32	19.67 ± 2.41	8.34 ± 2.15	5.39 ± 2.27
Right	10.13 ± 2.82	18.22 ± 3.67	19.58 ± 2.39	7.28 ± 2.01§	4.64 ± 2.29§
L_5_S_1_					
Two‐sided	13.37 ± 4.09‡	22.05 ± 3.96‡	20.40 ± 3.56‡	7.56 ± 2.41‡	3.86 ± 1.93‡
Left	13.29 ± 3.86	22.47 ± 3.86	20.71 ± 3.47	7.89 ± 2.48	3.88 ± 2.05
Right	13.46 ± 4.32	21.64 ± 4.04§	20.08 ± 3.64	7.23 ± 2.31§	3.84 ± 1.81
*F‐*value	124.85	255.85	7.25	11.03	43.35
*P*‐value	< 0.01	< 0.01	< 0.01	< 0.01	< 0.01

Notes: The two‐sided average value SNK‐*q* test shows that compared with L_3‐4_;

* *P* < 0.01; compared with L_4‐5_;

† *P* < 0.01; compared with L_5_S_1_;

‡ *P* < 0.01. The paired *t*‐test shows that compared with the left side;

§ *P* < 0.05

**Table 2:** Measurement on the rotating coronal plane (2) (mm, $\bar{x}$ *± s*)

Level/Side	W	S
L_2‐3_		
Two‐sided	10.87 ± 2.20	98.43 ± 29.00
Left	10.87 ± 2.20	99.47 ± 28.89
Right	10.86 ± 2.21	97.40 ± 29.23
L_3‐4_		
Two‐sided	12.19 ± 2.09*	96.76 ± 30.39
Left	12.16 ± 1.96	98.28 ± 31.66
Right	12.22 ± 2.22	95.24 ± 29.13
L_4‐5_		
Two‐sided	15.57 ± 2.38†	122.46 ± 41.72†
Left	15.70 ± 2.36	131.93 ± 43.72
Right	15.44 ± 2.38	112.99 ± 37.48§
L_5_S_1_		
Two‐sided	19.49 ± 2.63‡	148.12 ± 53.68‡
Left	19.38 ± 2.56	154.02 ± 55.38
Right	19.59 ± 2.70	142.22 ± 51.51§
*F*‐value	299.90	40.22
*P*‐value	< 0.01	< 0.01

Notes: The two‐sided average value SNK‐*q* test shows that compared with L_3‐4_;

* *P* < 0.01; compared with L_4‐5_;

† *P* < 0.01; compared with L_5_S_1_;

‡ *P* < 0.01. The paired *t*‐test shows that compared with the left side;

§ *P* < 0.05

**Table 3:** Measurement in the axial plane (mm, $\bar{x}$ *± s*)

Level/Side	Gs	Gi
L_2‐3_		
Two‐sided	9.13 ± 2.20	15.15 ± 2.54
Left	9.24 ± 2.24	15.18 ± 2.45
Right	9.01 ± 2.17	15.12 ± 2.64
L_3‐4_		
Two‐sided	7.94 ± 2.01*	13.81 ± 2.42*
Left	7.99 ± 2.05	13.86 ± 2.54
Right	7.89 ± 1.97	13.76 ± 2.31
L_4‐5_		
Two‐sided	8.47 ± 2.39	16.69 ± 2.65†
Left	8.58 ± 2.38	16.88 ± 2.68
Right	8.35 ± 2.41	16.50 ± 2.63
L_5_S_1_		
Two‐sided	10.60 ± 3.16‡	20.15 ± 3.65‡
Left	10.58 ± 3.00	20.10 ± 4.01
Right	10.62 ± 3.33	20.21 ± 3.25
*F*‐value	23.73	100.60
*P*‐value	< 0.05	< 0.05

Notes: The two‐sided average value SNK‐*q* test shows that compared with L_3‐4_;

* *P* < 0.01; compared with L_4‐5_;

† *P* < 0.01; compared with L_5_S_1_;

‡ *P* < 0.01. The paired *t*‐test shows that compared with the left side;

§ *P* < 0.05

### *Rotating Coronal Plane*

1. From the L_2‐3_ level to the L_5_S_1_ level, the distance of the exiting nerve root to the dural sac gradually increased at the superior/inferior margins of the intervertebral disc, and the inferior margin values were larger than the superior margin values (*P* < 0.05). At the superior margins of the intervertebral disc, the smallest value was measured at L_2‐3_ (6.71 ± 2.10 mm), and the largest was at L_5_S_1_ (13.37 ± 4.09 mm), improving by 6.66 mm. At the inferior margins, the smallest value was measured at L_2‐3_ (11.89 ± 2.55 mm), and the largest was at L_5_S_1_ (22.05 ± 3.96 mm), improving by 10.16 mm. Only the result for the left and right sides of the L_5_S_1_ level at the inferior margins was statistically significant, for which the difference value was 0.83 mm, as shown in Table 1.
2. For L_2‐3_ to L_5_S_1_, the vertical distance of the exiting nerve root to the pedicle gradually decreases from medial to lateral for each level. The vertical distance (Pa) from the exiting nerve root to the media of the pedicle gradually increased (*F* = 7.25, *P* < 0.0001); the smallest value was measured at L_2‐3_ (18.62 ± 2.50 mm), and the largest was at L_5_S_1_ (20.40 ± 3.56 mm), improving by 1.78 mm. The vertical distance (Pb) from the exiting nerve root to the middle of the pedicle gradually decreased (*F* = 11.03, *P* < 0.0001); the largest value was measured at L_2‐3_ (9.05 ± 1.82 mm), and the smallest was at L_5_S_1_ (7.56 ± 2.41 mm), reducing by 1.49 mm. The vertical distance (Pc) from the exiting nerve root to the lateral of the pedicle also gradually decreased (*F* = 43.35, *P* < 0.0001); the largest value was measured at L_2‐3_ (7.15 ± 2.27 mm), and the smallest was at L_5_S_1_ (3.86 ± 1.93 mm), reducing by 3.29 mm, as shown in Table 1. For the left and right sides of L_4‐5_ and L_5_S_1_, the Pb/Pc result was statistically significant, and the difference values were 1.06 mm (Pb in L_4‐5_), 0.66 mm (Pb in L_5_S_1_), and 0.75 mm (Pc in L_4‐5_), respectively, as shown in Table 1.
3. The width of the pedicle gradually increased from L_2‐3_ to L_5_S_1_ (*F* = 299.90, *P* < 0.0001). The smallest value was measured at L_2‐3_ (10.87 ± 2.20 mm), and the largest was at L_5_S_1_ (19.49 ± 2.63 mm), improving by 8.62 mm. There were no significant differences between the left and right sides, as shown in Table 2.
4. In the target intervertebral disc, the area of the safe working zone, identified as a trapezoid bounded by the lower vertebral pedicle and the exiting nerve root, gradually increased from L_2‐3_ to L_5_S_1_. The minimum value was measured at L_3‐4_ (96.76 ± 30.39 mm^2^), from which the area increased (*F* = 40.22, *P* < 0.0001), and the maximum was at L_5_S_1_ (148.12 ± 53.68 mm^2^), improving by 51.36 mm^2^. There was a significant difference between the left and right sides at L_4‐5_ and L_5_S_1_; the difference values were 18.94 mm^2^ and 11.8 mm^2^, respectively, as shown in Table 2.

### *Axis Plane*

The shortest distance from the exiting nerve root to the osseous articular process/facet joint surface at the axis plane in the superior/inferior margins of the disc was assessed. From L_2‐3_ to L_5_S_1_, both distances gradually increased, and the inferior margin values were greater than the superior margins values. The smallest distance in the superior margins occurs at L_3‐4_ (7.94 ± 2.01 mm), and the largest is at L_5_S_1_ (10.60 ± 3.16 mm), improving by 2.66 mm. The smallest distance in the inferior margins is observed at L_3‐4_ (13.81 ± 2.42 mm), and the largest occurs at L_5_S_1_ (20.15 ± 3.65 mm), improving by 6.34 mm. There were no significant differences between the left and right sides for either distance, as shown in Table 3.

## Discussion

Percutaneous transforaminal endoscopic lumbar interbody fusion is a procedure that has recently been developed based on percutaneous endoscopic lumbar discectomy. Its advantages include less skeletal muscle damage, less blood loss, and shorter hospital stays. The procedure requires removal of the protrusion intervertebral disc tissue under endoscopy to achieve a good decompression, and then the endplate is scraped and placed in the interbody cage^14^. The approach is based on the Kambin triangle of the posterior intervertebral foramen. For the small working zone between the exiting nerve root and the dural sac, endoscopic fusion surgery is challenging. If the procedure is not performed accurately, it can lead to nerve root injury, cage migration, and difficulty in achieving ideal fusion^16^. Therefore, it is essential that surgeons are familiar with the anatomy of this work area and related data.

In previous studies, most measurements of nerve roots in the intervertebral foramen have been acquired from cadaver specimens^17^. Before the relevant data can be measured, the relevant areas must be exposed, which will inevitably destroy the intervertebral foramen and the tissues surrounding the nerve roots, resulting in errors of various degrees^18^. This study avoids interference with the nerve roots by measuring the relevant data on CT images.

The Js, Ji, Pa, Pb, Pc, W and S values measured in this study were determined for a rotating coronal plane (on which the exiting nerve root is visible). The complete exiting nerve root and dural sac can be observed at this level, which renders the data measurement standards more uniform and reliable. At the superior and lower margins of the intervertebral disc, the distance from the exiting nerve root to the dural sac (Js, Ji) gradually increased from the L_2‐3_ level to the L_5_S_1_ level, and the inferior margin distance is smaller than the distance for the superior margin of the disc. Guan *et al*.^19^ measured the distance from the dural sac to the medial margins of the exiting nerve root at the superior endplate of the lower vertebrae using MRI. By comparing the results of our study, the data for L_2‐3_ and L_3‐4_ are significant; there is also a significant difference between the L_4‐5_ and L_5_S_1_ levels. This difference may arise because the MRI measurement is performed in the anterior and posterior coronal plane, but this study is based on the rotating coronal plane (the vertical plane of the long axis of the pedicle). The long axis of the pedicle has a certain angle with the sagittal plane (i.e. the pedicle inclination angle); the angle is smaller at the L_2‐3_ and L_3‐4_ levels and larger at the L_4‐5_ and L_5_S_1_ levels, which results in the difference between the results of Guan *et al*. and the present findings. According to the results of this study, the distance between the exiting nerve roots to the dural sac is smaller at the L_3‐4_ and L_2‐3_ levels. Therefore, when inserting a 10‐mm‐wide cage, the instruments can easily touch the exiting nerve root; thus, the surgeon should consider this anatomical distance. From the L_2‐3_ level to the L_5_S_1_ level, the vertical distance from the exiting nerve root to the pedicle gradually decreases from the medial side to the lateral border of the pedicle. The distance from the exiting nerve root to the middle and lateral border of the pedicle decreases from the L_2‐3_ level to the L_5_S_1_ level, especially the distance in the L_5_S_1_ levels with a minimum distance of 3.86 mm. Hardenbrook *et al*.^20^ measured the shortest distance from the exiting nerve root to the lateral border of the pedicle on a cadaver specimen and found a minimum value of 0.39 cm, which is consistent with the data in this study. Wang *et al*.^21^ measured the vertical distance from the ganglion tangential line to the vertebral pedicle in a cadaver specimen, with a value of approximately 8 mm, which is consistent with the distance from the exiting nerve root to the middle of the pedicle measured in this study. Because of this anatomical feature, it is easy to damage the exiting nerve root when inserting the working cannula and the interbody fusion cage, which should be brought to the attention of the surgeon. There were no significant differences between the left and right sides of the measured data at the L_2‐3_ or L_3‐4_ levels, but there were some significant differences at the L_4‐5_ and L_5_S_1_ levels, perhaps because the patients selected in this study were not control volunteers and had a larger age range.

The approach of minimally invasive endoscopic lumbar fusion surgery is based on the Kambin triangle^15^, while the actual operating area is an irregular shape. However, calculating the irregular shape area is complicated, and the channel is circular. To facilitate the calculation and further exploration of the area of this shape, Hardenbrook *et al*.^20^ proposed the concept of a safe area as the trapezoidal area enclosed by the superior and lower pedicles of the target intervertebral disc and the exiting nerve root. Following this method, we explored the approximately trapezoidal area in CT images. Because the values were measured in the same plane, the pedicle width and the abovementioned distance from the exiting nerve root to the middle of the pedicle can be utilized to calculate the area of the safe working zone. The area gradually increases for the L_2‐3_ to L_5_S_1_ levels, with a minimum of 0.97 cm^2^ for the L_3‐4_ level and a maximum of 1.48 cm^2^ for the L_5_S_1_ level. The area of the safe working zone calculated by Hardenbrook *et al*. also gradually increases from the L_2‐3_ level to the L_5_S_1_ level, with a maximum of 1.26 cm^2^ at the L_5_S_1_ level, which is smaller than the area calculated in this study. The reason for this discrepancy may be, first, that the measurement of Hardenbrook *et al*. was determined for a cadaver specimen. The formalin fixation and damage to the relevant structure during the anatomical process may have caused a difference in the calculated area. Second, the safe working zone in this study was calculated for the rotating coronal plane in which the nerve roots are visible on the long axis of the vertical pedicle, while the coronal plane was used for the cadaver specimen. Mirkovic *et al*.^22^ selected a triangle surrounded by the pedicle tangential line and the exiting nerve root in the anatomical coronal plane of a cadaver specimen to determine the optimum dimensions of the working cannula, with a maximum diameter of 9.7 mm at the L_5_S_1_ level. The calculated safe working zone provides a reference for percutaneous transforaminal endoscopic lumbar interbody fusion. The average area and the distance between the exiting nerve root to the lateral border of the pedicle are small; thus, the surgeon must be careful when performing related procedures, such as placement of the interbody cage. However, the calculated area of this safe working zone still has certain limitations. The shape of the trapezoid differs from the actual operating environment of the channel; thus, further optimization is needed for the measurement scheme. The difference between the left and right sides of the safe working zone area for L_4‐5_ and L_5_S_1_ is primarily due to the difference in the vertical distance from the exiting nerve root to the middle of the pedicle.

In the intervertebral foramen, the superior space is larger and corresponds to the position of the exiting nerve root exiting, and the lower part is narrow due to the presence of the small joint; however, this area has no important structures, so it can be removed during the endoscopic operation^14^. Removing parts of the superior/anterior lateral border of the facet joint could provide more room for the operation, facilitating placement of the working cannula and the interbody fusion cage and reducing damage to the nerve roots^23^. Our group measured the distance from the nerve root to the lateral border of the superior facet on the MRI axis in the superior and inferior margins of the intervertebral disc. From the L_2‐3_ level to the L_5_S_1_ level, the maximum value is 6.41 mm^24^. In this study, the distance from the exiting nerve root to the facet joint surface, which is equivalent to the removal of the entire superior articular process during surgery, on the CT axis was larger on the inferior margins of the disc. At the L_3‐4_ level, the Gs value is the smallest, with an average value of approximately 8 mm. After removal of the superior facet process, it is safe to place an 8‐mm working channel, but it is difficult to place a 10‐mm or larger working channel, which will affect the placement of a larger cage. Min *et al*.^25^ measured the distance from the exiting nerve root to the lateral border of the superior articular process at the inferior margins of the disc, but this distance was not the smallest; thus, the measured value is larger than the value obtained in this study. When performing percutaneous transforaminal endoscopic lumbar fusion, if the superior articular process can be properly removed, the safe working zone can be enlarged, and damage to the exiting nerve root can be reduced.

In 2012, Osman^13^ reported on percutaneous transforaminal decompression, interbody fusion, and percutaneous pedicle screw fixation for patients with degenerative disc disease and lumbar spondylolisthesis (ETDIF). The surgical approach is considered to be a natural progression of minimally invasive discectomy under endoscopy, and the patient’s postoperative outcome is better. However, Osman reported that although the surgical procedure is relatively simple, the working zone bounded by the traversing nerve root and the exiting nerve root is smaller, which is a limiting factor for widespread use. Hence, further prospective randomized controlled trials are needed to confirm the rationale of the procedure. Lee *et al*.^26^ performed percutaneous transforaminal endoscopic lumbar interbody fusion (PELIF) with an expandable spacer. In these cases, patients who had previously undergone lumbar surgery showed significant improvement; thus, the authors suggested that PELIF is more suitable for revision surgery. In addition, the single‐cage implant used in this procedure may be the cause of cage migration, subsidence, and collapse of the intervertebral space, and the authors propose that additional posterior fixation is a better choice for patients requiring revision surgery. This study is limited in that the sample size is too small and the procedure is not compared with other minimally invasive procedures; thus, further study of its applicability is needed. Wang and Grossman^27^ used an endoscopic technique for interbody fusion combined with percutaneous screw fixation and careful monitoring. Patients showed significant improvement, and no clear nonunion was found by radiography at the 1‐year follow‐up. However, its limitations also include a small sample size, short follow‐up time, and lack of a control group. Second, the indication of this procedure is limited; for example, this procedure may not be suitable for patients with severe bilateral symptoms or central spinal stenosis. Therefore, technological advances are needed to expand the spectrum of this minimally invasive procedure. Yao *et al*.^28^ reported that percutaneous endoscopic lumbar fusion surgery has a steep learning curve, requiring the surgeon to be familiar with the anatomy of the foraminal and to proficiently and safely perform endoscopic procedures. Jacquot and Gastambide^16^ performed the same percutaneous microscopic interbody fusion, and the authors reported that the incidence of postoperative complications (nerve root injury and cage migration) was 36%; they argued that this surgical approach should not be recommended unless there are major technical improvements.

Although the correlation distance from the exiting nerve root to the surrounding structure measured in the plane chosen in this work is different from that of the actual plane, the results of this study can still provide a reference for preoperative planning. However, the age range of this study is large, which may lead to measurement errors. Moreover, errors may also arise due to uncertainty in the density of the nerve root on the CT and the surrounding tissue density.

### *Conclusion*

The distance from the exiting nerve root to the surrounding tissue structure, as measured on a CT image, provides a reference for transforaminal endoscopic lumbar interbody fusion and for the size of the interbody cage in this procedure. It is more difficult to implant an interbody fusion cage with a width of 10 mm or more at the L_3‐4_ level and above; the removal of part of the superior articular process can enlarge the safe working zone and prevent damage to the nerve and surrounding important structures, which contributes to the placement of the cage. Therefore, surgeons must have a thorough understanding of the nerve roots and anatomy in this area to safely and effectively perform percutaneous endoscopic lumbar interbody fusion.
